# Assessment and Risk Analysis of Nitrosamines in Sausages From Northern Iran

**DOI:** 10.1002/fsn3.70380

**Published:** 2025-06-05

**Authors:** Mohammad Sadegh Allahkhah, Mohammadhosein Movassaghghazani

**Affiliations:** ^1^ Faculty of Veterinary Medicine Shab. C., Islamic Azad University Shabestar Iran; ^2^ Department of Food Hygiene and Quality Control, Faculty of Veterinary Medicine Shab. C., Islamic Azad University Shabestar Iran

**Keywords:** high‐performance liquid chromatography, Iran, nitrosamine, risk assessment, sausage

## Abstract

This study aimed to assess the levels of nitrosamines in sausages consumed in northern Iran and the associated health risks for consumers. For this purpose, two types of sausages, chicken and red meat (60 samples each), were randomly collected from meat product supply centers in the cities of Gilan province from December 2022 to March 2023. The levels of nitrosamines were determined using high‐performance liquid chromatography (HPLC). For risk evaluation, BMDL10 and MOE were used. In chicken sausages, nitrosamine levels ranged from 0.38 μg/kg (NDPA) to 7.77 μg/kg (NMEA). Red meat sausages ranged from 0.69 μg/kg (NDBA) to 9.59 μg/kg (NMEA). According to the results of the statistical analysis, the level of NDBA in the two types of sausage samples did not show a significant difference. However, a significant difference was observed in the mean levels of other nitrosamines between the two types of sausages. Red meat sausages had the highest mean nitrosamine concentration at 30.71 ± 0.87 μg/kg. The MOE values for both sausage types were below 10,000, indicating a health risk for consumers. These findings indicate the need for regulatory intervention to control nitrite levels in sausages in Gilan province.

## Graphical abstract

This study evaluates nitrosamine levels in chicken and red meat sausages from northern Iran, identifying health risks for consumers. Using high‐performance liquid chromatography (HPLC), significant variations in nitrosamine concentrations were observed, with red meat sausages showing the highest levels. The findings highlight potential health concerns and emphasize the need for regulatory action to control nitrite levels in processed meats.

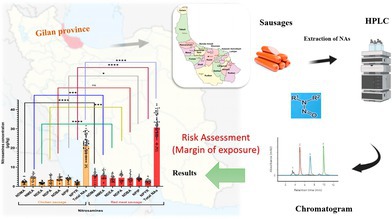

## 1 Introduction

Nitrate or nitrite is used for the preservation of food and is widely applied in meat products worldwide. This substance is added to meat products to prevent the growth and toxin production of *Clostridium botulinum* . In addition to these benefits, nitrate or nitrite also improves the color and flavor of meat products. Despite these numerous advantages, the consumption of this substance in food poses health risks for consumers. It seems that controlling the daily intake of this substance in food is very important for maintaining consumer health. Nitrite reacts with secondary amines in meat products, such as sausages, producing nitrosamines (NAs). NAs are a general term used for a large group of nitroso compounds with a chemical structure represented as R^1^N(R^2^)‐*N*=O (De Mey et al. 2014; Wexler and Anderson 2005; Nabizadeh et al. 2024). These compounds are classified into two groups: nitrosamines and nitrosamides. According to the classification by the International Agency for Research on Cancer (IARC), NAs are considered carcinogenic substances. Multiple studies indicate that the risk of gastrointestinal cancers increases due to NAs (De Mey et al. 2017; Houra et al. 2020; Ramezani et al. 2014). NAs can enter the liver through the bloodstream and are rapidly metabolized, showing their carcinogenic effects through DNA damage (De Mey et al. 2017; Houra et al. 2020; Ramezani et al. 2014). Therefore, the consumption of nitrite‐containing meat products due to the presence of NAs poses a health risk to consumers. The mechanisms linking the consumption of red and processed meats to cancer are not yet fully understood. N‐NAs (N‐nitrosamines) are produced in meat products containing nitrite or nitrate. NAs are a large group of compounds, many of which are carcinogenic. Therefore, the presence of NAs in processed meats may explain the public's hesitation to use meat products instead of red meat (De Mey et al. 2017). NAs are classified into volatile and non‐volatile types. Fifteen types of NAs have been identified in processed meats such as sausages, salami, and bacon. The type and level of NAs in meat products vary depending on the type of meat, amino precursors, carbonyls, storage conditions, cooking, and processing conditions (Nabizadeh et al. 2024). The most important volatile NAs recognized as carcinogenic by IARC include N‐Nitrosodimethylamine (NDMA), N‐Nitrosomethylethylamine (NMEA), N‐Nitrosodiethylamine (NDEA), N‐Nitrosodipropylamine (NDPA), N‐Nitrosodibutylamine (NDBA), N‐nitrosopiperidine (NPIP), and N‐Nitrosopyrrolidine (NPYR) (Ramezani et al. 2014).

According to the Iranian Statistical Center, in 2023, each Iranian's average per capita consumption of meat products is approximately 3.2 kg, which amounts to 8.76 g/day. The consumption of these products is particularly prevalent among younger individuals in Iran, and with the increased use of fast foods, the consumption rate has risen, contributing to a larger share of the Iranian diet (MPPSO 2024). The permissible limit of nitrite in heat‐treated meat products, such as sausages in Iran, is 120 mg/kg (ISIRI 2010). There is no standard for the permissible limit of NAs in meat products like sausages in Iran. Limited studies have been conducted on the levels of NAs in meat products such as sausages in Iran. In a study by Ramezani et al., the levels of NAs in 18 samples of heat‐treated meat products produced in Tehran were measured, with total nitrosamine levels ranging from 0.465 to 195 ng/g (Ramezani et al. 2014).

In a study conducted in South Korea, Lee et al. demonstrated that the highest levels of volatile NAs in fried, salted, smoked, cooked, and grilled meat products included N‐nitrosodimethylamine (NDMA), N‐nitrosopyrrolidine (NPYR), and N‐nitrosopiperidine (NPIP). The lowest levels were related to N‐nitrosodibutylamine (NDBA), N‐nitrosodiethylamine (NDEA), N‐nitrosomethylethylamine (NMEA), N‐nitrosomorpholine (NMOR), and N‐nitrosodipropylamine (NDPA). High temperatures consistently increase the rate of nitrosamine formation. Cooking methods such as frying and grilling lead to higher levels of NAs in meat products (Lee et al. 2024).

Volatile NAs are those that have significant carcinogenic activity, with NDEA being the strongest among them. It has been proven that low doses of NAs have carcinogenic effects on laboratory animals, which is why health authorities are concerned about the use of nitrite in meat products (Flores et al. 2019). The formation of NAs at high temperatures has been assessed in model systems to determine how various compounds may act as precursors. Thus, NDMA can be formed from several amino compounds such as glycine, choline, lecithin, sarcosine, creatine, creatinine, and betaine. Alanine can also produce NDEA in model systems, while proline, putrescine, and spermidine can produce NPYR (Flores et al. 2019).

The objective of this study was to determine the levels of volatile NAs in samples of chicken sausage and red meat sausage distributed in Gilan province in northern Iran using high‐performance liquid chromatography. Additionally, the risk of sausage consumption for consumers in Gilan province due to the presence of NAs was assessed using BMDL10 and MOE. Based on the evaluation of available resources, this research represents the inaugural study of its kind to be carried out in northern Iran. To date, no analogous studies have been conducted within this region.

## 2 Materials and Methods

### 2.1 Chemicals and Standards

Nitrosamine standards were purchased from Sigma Aldrich. Sodium hydroxide, ethanol, dichloromethane, sodium sulfate, and methanol were HPLC grade from Merck (Darmstadt, Germany).

### 2.2 Sample Collection Method

In this study, a total of 120 sausage samples, including 60 samples of sausage containing 60% chicken meat (four brands) and 60 samples of sausage containing 60% red meat (four brands), were randomly collected from supply centers in five cities of Gilan province (including Rasht, Anzali, Astaneh, Fuman, and Lahijan) from December 2022 to March 2023. All sausage samples were heat‐treated products. For sampling, each city was divided into five regions: north, south, east, west, and center. The supply centers were marked on a map, and samples were collected from each area as needed. The samples were sent to the laboratory under sterile conditions and in the presence of dry ice, and they were stored at −20°C until chromatographic analyses were performed.

Iranian sausages, commonly referred to as “Sosis” in Persian, are a popular processed meat product in Iran. They are characterized by their smooth, emulsified texture and mild flavor. Typically made from a blend of meats (often beef or chicken) combined with spices, starch, and other ingredients, these sausages are processed into a fine paste and then encased. During production, the mixture is usually heated to approximately 70°C to ensure proper cooking and to achieve the signature firm texture.

### 2.3 Analysis of Sausage Samples by HPLC

#### 2.3.1 Sample Preparation

The levels of NDMA, NMEA, NDEA, NDPA, NDBA, NPIP, and NPYR were determined by HPLC in sausage samples. Initially, 6 g of the sausage sample was placed in a Pyrex tube, and 10 mL of 1 N sodium hydroxide was added. The solution was transferred to a 50 mL separating funnel. The tubes were rinsed twice with 5 mL of ethanol and then with 10 mL of dichloromethane. The rinsing solutions were combined with 10 mL of a 10% sodium chloride aqueous solution and the original sample in the separating funnel. After shaking, the dichloromethane layer was collected, and the aqueous layer was re‐extracted with 10 mL of dichloromethane. The extracted dichloromethane was dried using anhydrous sodium sulfate to remove moisture. This extracted solution was then concentrated to about 0.5 mL using a Kuderna‐Danish (KD) apparatus and nitrogen gas flow (Al‐Kaseem et al. 2014).

The concentrated solution was loaded onto a silica gel column (30 cm × 1.5 cm), and the column was washed with 10 mL of dichloromethane. After adding 100 μL of octane (to prevent complete evaporation of the solvent), the washing solution was again concentrated using the KD apparatus and nitrogen gas flow to reach a volume of 1 mL, after which 3 mL of methanol was added for extraction. This process was repeated three times. The methanol‐extracted materials were ultimately concentrated under nitrogen gas flow to about 100 μL (Al‐Kaseem et al. 2014).

#### 2.3.2 HPLC Conditions

A Unicam Crystal‐200 chromatography (England) was used. Chromatography was performed using a Knauer Symmetry C18 column, 5 μm (100 mm × 4.6 mm). Solvent A was a 10 mmol ammonium hydroxide solution, and acetonitrile was used as solvent B. This solution was filtered through a PVDF membrane filter with a pore diameter of 0.22 μm and degassed under a vacuum before use. Separation was carried out using a gradient wash of solvents A and B. The injection volume was 20 μL, with a gradient wash (solvent B from 0% to 90% over 10 min and then maintained until the end of the run), at a flow rate of 1 mL/min at a temperature of 80°C, with a detection wavelength of 231 nm and a sample temperature of 15°C (Al‐Kaseem et al. 2014).

### 2.4 Method Validation

#### 2.4.1 NA Standards

Calibration curves were created using standard solutions of seven NAs (NDMA, NMEA, NDEA, NDPA, NDBA, NPIP, and NPYR) in acetonitrile at levels ranging from 0.00 to 10 μg/kg and analyzed in triplicate. The concentrations of the NAs in the samples were correlated linearly with the integrated peak areas of the standards.

#### 2.4.2 Limits of Detection (LODs) and Quantification (LOQs)

LODs and LOQs were calculated for each analytical method using a signal‐to‐noise ratio of 3:1 and 10:1, respectively.

#### 2.4.3 Linearity

Linearity was confirmed in all chromatographic runs by checking the coefficient of determination (*r* ^2^) on the residual plots of the calibration curves.

#### 2.4.4 Specificity

The specificity of the method was evaluated by comparing the retention times in the blank sample matrices and the samples spiked with 5 μg/kg of NDMA and NMEA; 4 μg/kg of NDEA, NDPA, NDBA, NPIP, and NPYR to ensure there was no interference in the retention time of the target analytes.

#### 2.4.5 Matrix Effects

To investigate matrix effects, samples of chicken sausage and red meat sausage available in the local market were selected. The magnitude of matrix effects was estimated by comparing the slopes of solvent (bsol) and matrix‐matched calibrations (bmm) and quantitatively expressed as the signal suppression/enhancement (SSE) ratio using the following equation:

$$\mathrm{SSE}\;\left(\%\right)=\frac{\mathrm{bmm}-\text{bsol}}{\text{bsol}}\times 100$$

#### 2.4.6 Precision

The precision level, represented as relative standard deviations (RSDs), was evaluated by performing intra‐day repeatability and inter‐day reproducibility using spiked blank samples with nitrosamine standard solutions at four concentration levels (2, 4, 6, and 8 μg/kg) for NDMA, NMEA, NDBA, NPIP, and NPYR and three concentration levels (2, 4, and 6 μg/kg) for NDEA and NDPA.

### 2.5 Risk Evaluation

#### 2.5.1 Daily Exposure Level

To determine the Estimated Daily Intake (EDI), the following formula was used. According to the latest statistics, the per capita sausage consumption in Iran is 0.000876 kg/person/day. An average weight of 70 kg was considered for adults.

$$\mathrm{EDI}=\frac{\text{Mean of}\;\mathrm{NAs}\;\text{in sausages}\;\left(\mathrm{\mu g}/\mathrm{kg}\right)\times \text{daily sausages intake}\;\left(\mathrm{kg}/\mathrm{day}\right)}{\text{Average body weight}\;\left(\mathrm{kg}\right)}$$

#### 2.5.2 The Margin of Exposure (MOE)

The MOE is the ratio of the level of exposure for humans to the lower confidence limit of the benchmark dose of 10% (BMDL10), which is derived from animal toxicity studies. The results are obtained by dividing the exposure by the toxicological reference (BMDL), where BMDL10 (rat) = 10 μg/kg of body weight per day (EFSA 2023). The MOE was calculated by using the following equation:

$$\mathrm{MOE}=\frac{\text{BMDL}10}{\mathrm{EDI}}$$

### 2.6 Data Analysis Method

A *t*‐test was used to compare nitrosamine levels between the two types of sausages. The *t*‐test was also employed to compare the total nitrosamine levels between the two types of sausages. All statistical tests were performed using SPSS version 26 and GraphPad Prism 10.2.1 (GraphPad Software, Boston, Massachusetts, USA). A significance level of 0.05 was considered.

## 3 Results and Discussion

### 3.1 The Mean of NAs in Sausage Samples

**FIGURE 1:**
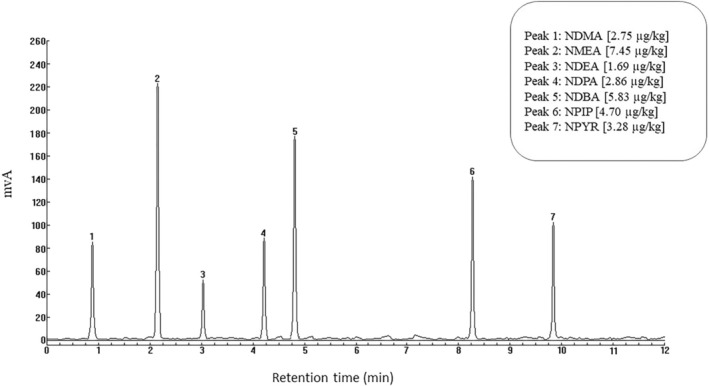
Chromatogram of nitrosamine levels in chicken sausage samples.

**FIGURE 2:**
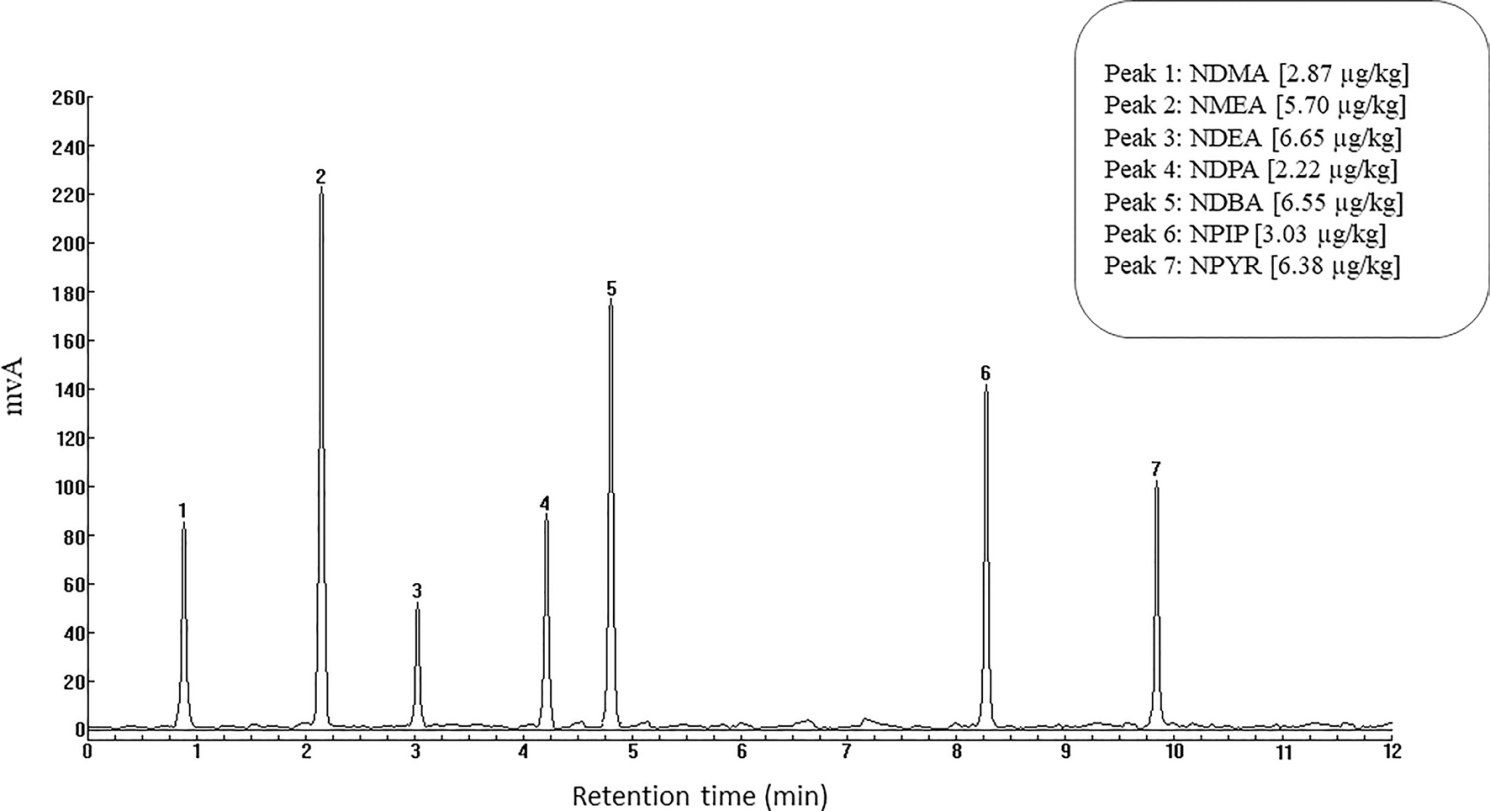
Chromatogram of nitrosamine levels in red meat sausage samples.

The chromatograms of the samples related to the levels of NDMA, NMEA, NDEA, NDPA, NDBA, NPIP, and NPYR in two types of sausages in Gilan province are shown in Figures 1 and 2.

Table 1 shows the mean of NAs in sausage samples. In chicken sausage samples, the lowest level of nitrosamine was related to NDPA at 0.38 μg/kg, while the highest was related to NMEA at 7.77 μg/kg. In red meat sausage samples, the lowest nitrosamine level was related to NDBA at 0.69 μg/kg, and the highest was related to NMEA at 9.59 μg/kg.

**TABLE 1:** The comparison of the mean of NAs in two types of sausage samples in Gilan province.

NAs	Mean ± SE (μg/kg)		Min (μg/kg)		Max (μg/kg)		*p*
	Chicken	Red meat	Chicken	Red meat	Chicken	Red meat	
NDMA	2.40 ± 0.14	5.57 ± 0.26	0.68	0.73	5.39	9.50	< 0.0001
NMEA	4.37 ± 0.23	5.60 ± 0.25	0.60	1.53	7.77	9.59	0.001
NDEA	1.78 ± 0.07	4.23 ± 0.20	0.78	1.91	3.31	7.52	< 0.0001
NDPA	3.28 ± 0.24	4.50 ± 0.26	0.38	1.12	6.83	8.46	0.001
NDBA	3.87 ± 0.15	4.13 ± 0.26	1.07	0.69	6.55	8.86	0.410
NPIP	3.59 ± 0.14	4.02 ± 0.10	0.90	2.49	5.92	6.38	0.020
NPYR	1.82 ± 0.09	2.62 ± 0.13	0.44	0.84	3.51	5.57	< 0.0001
Total	21.15 ± 0.72	30.71 ± 0.87	9.76	18.38	31.62	42.95	< 0.0001

*Note:* Number of samples for each type of sausage: 60.

Abbreviation: SE, standard error.

According to the results of the statistical analysis, the level of NDBA did not show a significant difference between the two types of sausage samples (*p* > 0.05). A significant difference was observed in the average levels of other NAs between the two types of sausages (*p* < 0.05). Regarding the average total NAs, the highest level was found in the red meat sausage sample at 30.71 ± 0.87 μg/kg (*p* < 0.05).

**FIGURE 3:**
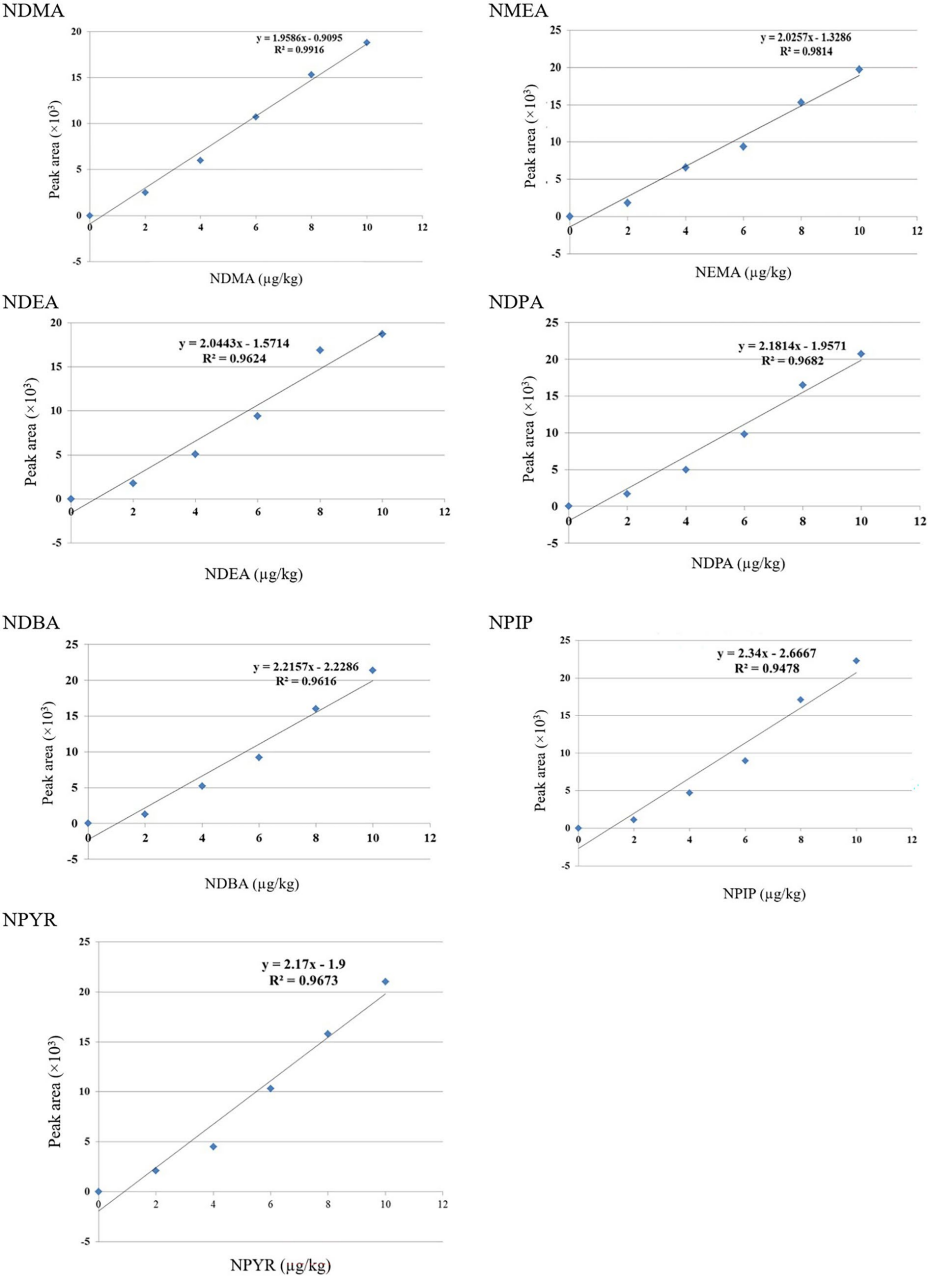
The calibration curves of NAs.

**FIGURE 4:**
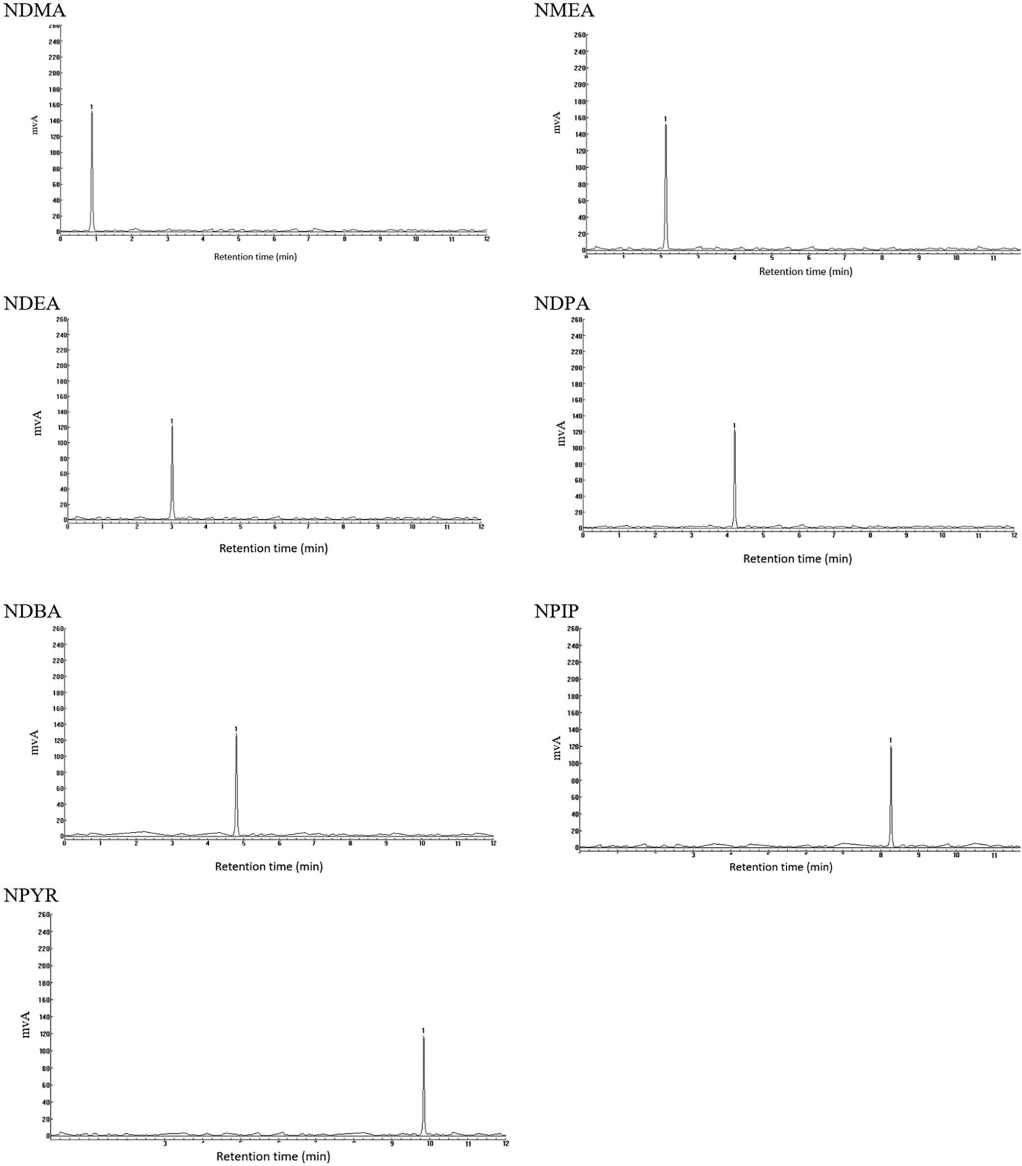
The blank sausage samples were spiked with NAs.

Tables 2 and 3 indicate the validation settings for determining NAs in sausages using HPLC. The calibration curve showed that the procedure was linear (*r* ^2^ coefficient of determination for NAs: NDMA = 0.9916, NMEA = 0.9814, NDEA = 0.9624, NDPA = 0.9682, NDBA = 0.9616, NPIP = 0.9478, and NPYR = 0.9673) (Figure 3). A graph validated the method's specificity spiked with 5 μg/kg of NDMA and NMEA and 4 μg/kg of NDEA, NDPA, NDBA, NPIP, and NPYR, ensuring no interaction with the target analyte's retention time (Figure 4). The percent extraction recovery (ER%) ranged from 87.44 to 105.17 (Table 2). According to Table 3, the matrix effects in terms of SSE for HPLC of the target chemicals in various matrices were positive, showing signal augmentation. RSD values were ≤ 5.54%, both intra‐day and inter‐day. High recovery rates demonstrated that matrix effects were not significant in this investigation (Table 3). Furthermore, the absence of any signal response near nitrosamine retention duration in all matrices suggested that no matrix interferences existed, despite the matrices' great complexity.

**TABLE 2:** Validation parameters for determining NAs in sausages by the HPLC method.

Type of sausage	Spiked level (μg/kg)	Intra‐day (*n* = 3) RSD (%)							Inter‐day (*n* = 4) RSD (%)							ER (%)						
		NDMA	NMEA	NDEA	NDPA	NDBA	NPIP	NPYR	NDMA	NMEA	NDEA	NDPA	NDBA	NPIP	NPYR	NDMA	NMEA	NDEA	NDPA	NDBA	NPIP	NPYR
Chicken	2	5.17	2.95	4.36	2.66	5.40	2.50	2.71	3.57	3.69	5.04	4.91	3.81	4.81	3.52	103.20	97.51	92.81	88.61	96.60	93.36	105.17
	4	3.86	4.57	3.79	5.03	4.35	4.06	4.49	4.62	5.46	2.91	3.66	5.20	3.30	4.93	95.44	94.33	101.50	103.20	105.31	104.41	98.35
	6	4.19	3.32	5.25	4.18	3.81	3.35	4.76	5.04	3.89	4.20	3.10	4.03	4.47	3.21	89.17	105.11	95.35	94.11	92.83	88.52	91.40
	8	3.30	5.17	—	—	5.03	4.70	5.35	4.46	3.11	—	—	4.74	5.11	4.60	93.31	90.60	—	—	90.16	95.07	90.07
Red meat	2	4.63	4.82	4.81	2.70	2.70	4.02	3.24	2.94	5.30	4.41	4.40	4.88	3.07	2.71	90.66	102.04	88.61	90.67	87.41	100.60	93.56
	4	5.54	3.93	3.69	3.61	3.15	3.20	2.90	4.33	4.44	4.90	4.80	3.53	3.79	3.96	94.17	91.80	96.16	105.21	98.54	94.12	102.70
	6	3.21	3.24	2.70	5.27	3.81	5.07	3.81	3.70	4.06	5.41	3.54	5.20	4.55	4.34	96.82	95.22	97.80	93.07	94.40	91.50	87.44
	8	4.16	2.91	—	—	4.50	4.18	4.79	5.37	3.55	—	—	3.13	4.88	5.15	104.5	88.34	—	—	90.59	104.69	89.61

*Note:* Relative standard deviations for intra‐ and inter‐day precision at concentrations of 2, 4, 6, and 8 μg/kg of NAs, except for NDEA and NDPA (2, 4, and 6 μg/kg), were calculated.

Abbreviations: ER, extraction recovery; RSD, relative standard deviations.

**TABLE 3:** The results of the validation method for LODs, LOQs, and matrix effects for determining NAs in sausages by the HPLC method.

Type of sausage	Matrix effect‐SSE (%)							LOD (μg/kg)							LOQ (μg/kg)						
	NDMA	NMEA	NDEA	NDPA	NDBA	NPIP	NPYR	NDMA	NMEA	NDEA	NDPA	NDBA	NPIP	NPYR	NDMA	NMEA	NDEA	NDPA	NDBA	NPIP	NPYR
Chicken	5.5	3.3	3.9	3.1	2.5	3.5	3.8	0.026	0.081	0.009	0.007	0.028	0.036	0.046	0.061	0.106	0.025	0.010	0.115	0.040	0.071
Red meat	4.6	2.8	4.8	4.3	4.0	4.6	4.9	0.013	0.021	0.017	0.029	0.009	0.014	0.024	0.077	0.040	0.033	0.063	0.050	0.019	0.052

Abbreviations: LOD, limit of detection (S/*N* = 3); LOQ, limit of quantification (S/*N* = 10); SSE, signal suppression/enhancement.

Moradi et al., in a study conducted in Tehran, determined the levels of NAs in 150 samples of red meat and chicken sausages using chromatography in 2018. In the red meat sausage samples, the levels of NPYR, NPIP, and total NAs were higher than those in chicken sausage samples. The current study did not show a significant difference in NDBA levels between the two types of sausages (Moradi et al. 2021).

The results of the current study indicated that the total nitrosamine levels in red meat sausage were higher than in chicken sausage. The findings of the current study were similar to those of Moradi and colleagues. It seems that the type of meat composition affects the nitrosamine levels in the final product.

In other studies, the role of various cooking methods, fat content, meat content, and moisture in the formation of NAs has been demonstrated (De Mey et al. 2017).

Ramazani et al. determined the levels of NAs in sausage samples in a study conducted in Tehran. The lowest and highest levels of NAs were 0.465 and 195 μg/kg, respectively. The highest nitrosamine levels were observed in samples containing 90% meat (Ramezani et al. 2014).

Alizadeh et al. examined nitrite levels in sausage samples in a study conducted in Kerman, Iran. The nitrite residues in 15.3% of the samples exceeded the permissible limits of the national standard of Iran. It seems that neglecting to adhere to the standard nitrite limits in produced sausages leads to increased nitrosamine levels in the final product (Alizadeh et al. 2015). In a study conducted in Mazandaran province, the levels of nitrate and nitrite were examined in 43 meat product samples. The nitrite levels in the sausage samples were within the permissible limits (Alizadeh et al. 2015).

A review of reports regarding nitrosamine content in processed meat products in the European Union has shown that the average NDMA content is less than 2.7 μg/kg, and NDEA is below 0.9 μg/kg. Thus, the total of NDMA and NDEA is less than 3.6 μg/kg, which is a very low amount (Flores et al. 2019).

In a study conducted in Craiova, Romania, the levels of some NAs in meat products were determined. In meat products, the NDMA levels ranged from 0.80 to 23.40 μg/kg, and the NDEA levels ranged from 11.60 to 61.90 μg/kg. According to the results of the current study, the levels of these two NAs were lower than those found in Romania (Houra et al. 2020).

Park et al. collected 387 different food samples over 3 years from seven major cities in South Korea and determined the nitrosamine levels in the samples using chromatography. The results showed that the NDMA levels in 28 fish sausage samples ranged from 0.2 to 2.71 μg/kg (Park et al. 2015).

De Mey et al. collected 101 samples of fermented sausages from four major stores in Belgium in 2011. The nitrosamine levels in the samples were determined using chromatography. N‐nitrosopiperidine and N‐nitrosomorpholine were identified in 22% and 28% of the samples, respectively. It seems that the formation of NPIP is related to the presence of piperidine, which originates from black pepper, in the formulation of products in Belgium. In the current study, NPIP was observed in both types of sausages in Gilan province, which, considering the use of black pepper in sausage production in Iran, could be one of the reasons for the observed NPIP nitrosamine (De Mey et al. 2017).

In research conducted in Spain, the researchers collected 47 different samples of processed meat products and determined the levels of volatile NAs in the samples using chromatography. The highest level of NAs in the samples was 5.4 μg/kg, while the lowest level of NAs was related to NPYR (Fernández et al. 2024).

In a study, Herrmann et al. examined the levels of NAs in processed meat products in Denmark and Belgium and investigated the effect of heat on NAs. In this study, 70 samples from Danish products and 20 samples from Belgian products were collected. Among the collected samples, the total volatile nitrosamine levels exceeded 10 μg/kg in only one Danish sample and two Belgian samples, which were significantly lower than the total nitrosamine levels found in the current study in Iran. During the cooking and frying processes, NPIP was detected, but the levels of other NAs varied depending on the product type and cooking process. The levels of non‐volatile NAs and N‐nitrosothiazolidine‐4‐carboxylic acid decreased during the cooking process (Herrmann et al. 2015).

According to studies conducted in other countries, it appears that the nitrite levels in sausage are one of the most important factors in determining the nitrosamine levels in the final product. However, for NDMA and NPYR, increasing nitrite levels does not affect their levels (Zhang et al. 2023). It has now been established that the use of substances such as ascorbic acid, alpha‐tocopherol, and erythorbic acid prevents the formation of NAs (Flores et al. 2019).

### 3.2 The MOE

Table 4 shows the results of the risk evaluation of NAs in different types of sausages in Gilan province. Based on the results, the MOE value for total nitrosamines in chicken and red meat sausages was below 10,000, suggesting a potential health risk for consumers. However, the MOE value for each nitrosamine exceeded 10,000, indicating no significant health risk. Based on the results, the level of NDBA did not show a significant difference between the two types of sausage samples (*p* > 0.05). A significant difference was observed in the average levels of other NAs between the two types of sausages (*p* < 0.05). Regarding the average total NAs, the highest level was found in the red meat sausage sample at 30.71 ± 0.87 μg/kg (*p* < 0.05). The MOE value in both chicken and red meat sausages was less than 10,000, indicating a health risk for consumers.

**TABLE 4:** Estimating risk assessment parameters for NAs in sausages from Gilan province, Iran.

Type of sausage	NAs	Daily consumption (kg/day)	Mean of NAs (μg/kg)	Average EDI (μg/kg/day)	Average MOE
Chicken	NDMA	0.00876	2.40	0.0003	33,333
	NMEA	0.00876	4.37	0.0005	20,000
	NDEA	0.00876	1.78	0.0002	50,000
	NDPA	0.00876	3.28	0.0004	25,000
	NDBA	0.00876	3.87	0.0004	25,000
	NPIP	0.00876	3.59	0.0004	25,000
	NPYR	0.00876	1.82	0.0002	50,000
	Total	0.00876	21.15	0.00264	3787
Red meat	NDMA	0.00876	5.57	0.0006	16,666
	NMEA	0.00876	5.60	0.0007	14,285
	NDEA	0.00876	4.23	0.0005	20,000
	NDPA	0.00876	4.50	0.0005	20,000
	NDBA	0.00876	4.13	0.0005	20,000
	NPIP	0.00876	4.02	0.0005	20,000
	NPYR	0.00876	2.62	0.0003	33,333
	Total	0.00876	30.71	0.00384	2604

*Note:* An average body weight of 70 kg was used for adults.

Abbreviations: EDI, estimated dietary intake; MOE, margin of exposure.

The MOE values for both types of sausages were below 10,000, indicating a potential health risk for consumers. This finding is particularly concerning given the carcinogenic nature of NAs. MOE is a critical parameter in risk evaluation, and values below 10,000 typically indicate the need for risk management measures to protect public health.

Moradi et al. in their risk evaluation of NAs in sausages distributed in Tehran showed that based on the levels of NPIP and NPYR in the collected samples and according to the method of the United States Environmental Protection Agency, sausage consumption in Tehran poses a carcinogenic risk below the health threshold. In the current study, considering the MOE values and the risks associated with sausage consumption in Gilan province, implementing risk management measures is essential for consumers (Moradi et al. 2021).

Due to differences in composition and processing, red meat sausages tend to have higher levels of N‐nitroso compounds (NAs) than chicken sausages. Red meat is rich in myoglobin and heme iron, which promote the formation of NAs during curing and cooking; additionally, red meat sausages tend to have higher fat content, which can further contribute to the production of NAs; additionally, the use of nitrites as preservatives is generally more common in the processing of red meat sausages, which creates precursors for NAs; and finally, red meat sausages are frequently cooked at higher temperatures, which accelerate the formation of NAs. In contrast, chicken sausages are less susceptible to these reactions because they are leaner and contain less heme iron (Zhang et al. 2023). The limitation of the study lies in the fact that it did not examine the levels of nitrite and nitrate. This aspect could provide more comprehensive insights and should be addressed in future investigations.

## 4 Conclusion and Future Perspective

Based on the results obtained, this study emphasizes the importance of continuous monitoring and oversight to ensure the safety of food products. The significant presence of NAs and associated health risks requires immediate attention from food safety authorities to implement appropriate control measures and safeguard consumer health in Gilan province. This study underscores the critical need for continuous monitoring and oversight to ensure food safety in Gilan province, particularly due to the significant health risks posed by the presence of NAs. Based on the results, the MOE value for total nitrosamines in both chicken and red meat sausages was below 10,000, suggesting a potential health risk for consumers. However, the MOE value for each nitrosamine exceeded 10,000, indicating no significant health risk. Food safety authorities need to enforce stricter regulations and standards for permissible nitrite levels in food products while also implementing robust training programs for food producers to raise awareness and encourage best practices to reduce NAs. Consumer education initiatives are equally important to inform the public about the potential health risks associated with these compounds. Collaborative efforts between local authorities, research institutions, and food production units could lead to innovative technologies to mitigate these risks. Future research should focus on long‐term studies to explore the public health impacts of NAs and nitrites, investigate safer preservation alternatives, and develop advanced detection methods for monitoring these substances. Expanding such studies to other provinces may help identify broader trends and support unified nationwide food safety measures. These efforts collectively aim to safeguard consumer health and enhance the quality and safety of processed foods.

## Author Contributions

**Mohammad Sadegh Allahkhah:** data curation (equal), investigation (equal). **Mohammadhosein Movassaghghazani:** conceptualization (equal), data curation (equal), formal analysis (lead), investigation (lead), methodology (lead), project administration (lead), validation (lead), writing – original draft (lead), writing – review and editing (lead).

## Conflicts of Interest

The authors declare no conflicts of interest.

## Data Availability

Data are available on request from the authors.
